# Evaluation of a combined triple method to detect causative HPV in oral and oropharyngeal squamous cell carcinomas: p16 Immunohistochemistry, Consensus PCR HPV-DNA, and In Situ Hybridization

**DOI:** 10.1186/1750-9378-7-4

**Published:** 2012-02-29

**Authors:** Giuseppe Pannone, Vito Rodolico, Angela Santoro, Lorenzo Lo Muzio, Renato Franco, Gerardo Botti, Gabriella Aquino, Maria Carmela Pedicillo, Simona Cagiano, Giuseppina Campisi, Corrado Rubini, Silvana Papagerakis, Gaetano De Rosa, Maria Lina Tornesello, Franco M Buonaguro, Stefania Staibano, Pantaleo Bufo

**Affiliations:** 1 Department of Surgical Sciences - Section of Anatomic Pathology and Cytopathology, University of Foggia, Viale Luigi Pinto 1, 71122 Foggia, Italy; 2 Dipartimento di Scienze per la promozione della Salute - Sez. Anatomia Patologica, Università degli Studi di Palermo, A.O.U. Policlinico "P. Giaccone" - Via del Vespro 129, 90127 Palermo, Italy; 3 Department of Surgical Sciences - Section of Oral Pathology, University of Foggia, Foggia, Italy; 4 Istituto Nazionale per lo Studio e la Cura dei Tumori - Fondazione 'G. Pascale', Naples, Italy; 5 Dipartimento di Scienze Stomatologiche, Università di Palermo, Via del Vespro 129, 90127 Palermo, Italy; 6 Sezione di Anatomia Patologica, Università Politecnica delle Marche, Ancona, Italy; 7 Department of Otolaryngology - Head and Neck Surgery - Laboratory of Oral, Head and Neck Cancer Invasion and Metastasis, Medical School, University of Michigan Ann Arbor, Ann Arbor, MI, USA; 8 Dipartimento di Scienze Biomorfologiche e Funzionali, Università degli Studi di Napoli 'Federico II', Via Sergio Pansini 5, 80131 Naples, Italy; 9 Laboratory of Molecular Biology and Viral Oncogenesis & AIDS Reference Center, Istituto Nazionale Tumori Fondazione G. Pascale, Via Mariano Semmola 1, 80131 Naples, Italy

**Keywords:** Head and neck squamous cell carcinoma, HN-SCC, OSCC, OPSCC, Human papillomavirus, HPV, DNA consensus PCR, Immunohistochemistry, IHC, p16-IHC, Epigenetic, Methylation-Specific PCR

## Abstract

**Background:**

Recent emerging evidences identify Human Papillomavirus (HPV) related Head and Neck squamous cell carcinomas (HN-SCCs) as a separate subgroup among Head and Neck Cancers with different epidemiology, histopathological characteristics, therapeutic response to chemo-radiation treatment and clinical outcome. However, there is not a worldwide consensus on the methods to be used in clinical practice. The endpoint of this study was to demonstrate the reliability of a triple method which combines evaluation of: 1. p16 protein expression by immunohistochemistry (p16-IHC); 2. HPV-DNA genotyping by consensus HPV-DNA PCR methods (Consensus PCR); and 3 viral integration into the host by in situ hybridization method (ISH). This triple method has been applied to HN-SCC originated from oral cavity (OSCC) and oropharynx (OPSCC), the two anatomical sites in which high risk (HR) HPVs have been clearly implicated as etiologic factors. Methylation-Specific PCR (MSP) was performed to study inactivation of p16-CDKN2a locus by epigenetic events. Reliability of multiple methods was measured by Kappa statistics.

**Results:**

All the HN-SCCs confirmed HPV positive by PCR and/or ISH were also p16 positive by IHC, with the latter showing a very high level of sensitivity as single test (100% in both OSCC and OPSCC) but lower specificity level (74% in OSCC and 93% in OPSCC).

Concordance analysis between ISH and Consensus PCR showed a faint agreement in OPSCC (κ = 0.38) and a moderate agreement in OSCC (κ = 0.44). Furthermore, the addition of double positive score (ISHpositive and Consensus PCR positive) increased significantly the specificity of HR-HPV detection on formalin-fixed paraffin embedded (FFPE) samples (100% in OSCC and 78.5% in OPSCC), but reduced the sensitivity (33% in OSCC and 60% in OPSCC). The significant reduction of sensitivity by the double method was compensated by a very high sensitivity of p16-IHC detection in the triple approach.

**Conclusions:**

Although HR-HPVs detection is of utmost importance in clinical settings for the Head and Neck Cancer patients, there is no consensus on which to consider the 'golden standard' among the numerous detection methods available either as single test or combinations. Until recently, quantitative E6 RNA PCR has been considered the 'golden standard' since it was demonstrated to have very high accuracy level and very high statistical significance associated with prognostic parameters. In contrast, quantitative E6 DNA PCR has proven to have very high level of accuracy but lesser prognostic association with clinical outcome than the HPV E6 oncoprotein RNA PCR. However, although it is theoretically possible to perform quantitative PCR detection methods also on FFPE samples, they reach the maximum of accuracy on fresh frozen tissue. Furthermore, worldwide diagnostic laboratories have not all the same ability to analyze simultaneously both FFPE and fresh tissues with these quantitative molecular detection methods. Therefore, in the current clinical practice a p16-IHC test is considered as sufficient for HPV diagnostic in accordance with the recently published Head and Neck Cancer international guidelines. Although p16-IHC may serve as a good prognostic indicator, our study clearly demonstrated that it is not satisfactory when used exclusively as the only HPV detecting method. Adding ISH, although known as less sensitive than PCR-based detection methods, has the advantage to preserve the morphological context of HPV-DNA signals in FFPE samples and, thus increase the overall specificity of p16/Consensus PCR combination tests.

## Background

Oral and oropharyngeal squamous cell carcinomas (OSCCs and OPSCCs, respectively) represent a major health issues, with over 200,000 new cases reported worldwide annually. Though improvements in screening and early diagnosis have dramatically reduced the incidence of these neoplasms in recent years, the 5-year disease-free survival is still poor, despite significant scientific and financial efforts. Recently, several studies have shown that HPV are clearly involved in the pathogenesis of a subgroup of OSCC and OPSCC [1,2]. This distinct subgroup of Head and Neck Cancers is characterized by distinctive histopathological features: HPV infection, distinctive epidemiology, better response to induction chemotherapy and concurrent chemo-radiation protocol and an overall better clinical outcome, as compared to HPV negative HN-SCC [3-6]. The proportion of OPSCCs that are potentially HPV-related (cancers of the tongue-based and tonsils, including lingual tonsil and Waldeyer's ring) increased in the USA from 1973 to 2004, perhaps as a result of changing sexual behaviours. Nevertheless, OPCCs associated with HPV infection show a better prognosis and seems to occur predominantly in unmarried younger patients ( < 40 yrs), especially males. Therefore, there is a need to properly assess OPSCC subgroups: 1) HPV-unrelated/classic OPCCs that are less responsive to conventional anti-cancer therapies; 2) HPV associated OPSCCs with less mortality and recurrence rates with mutiple management options. Since Syrjanen's initial observations in 1983 [7], there have been numerous reports on HPV-DNA detection in HN-SCC with rates varying from 0% to 100% of tumors studied [8,9]. These differences in detection rate are due to at least two principal factors: a) differences in the epidemiological distribution of oncogenic HR-HPVs in the world; b) different analytical methods utilized [10,11].

The p16INK4A gene functions as a negative regulator of the cell cycle progression through its inhibition of cdk4/6 which in turn determines the blockage of the cyclin-dependent phosphorylation of the Retinoblastoma protein (Rb). Loss of heterozygosity (LOH), hypermethylation, deletion, mutation of the p16INK4A locus are common events in Head and Neck carcinogenesis [12-16]. Therefore, p16INK4A expression loss defines a subgroup of OPSCC patients with worse clinical outcome [17]. Furthermore, as with female genital (or cervical) carcinogenesis, the immunohistochemical detection of p16 protein (p16-IHC) has been proposed as surrogate marker of HPV infection in Head and Neck Cancer [18]. However, although recent publication of Guidelines for Head and Neck Cancer [National Comprehensive Cancer Network (NCCCN) Guidelines™ Version 1.2011 Head and Neck Cancers] suggests p16-IHC as a screening method for HPV detection [19], some questions remain regarding the accuracy of the test when used alone, without molecular detection of HPV-DNA. The HPV-DNA test may be used in Head and Neck Pathology departments with the following diagnostic and prognostic purposes: a) distinguish HPV positive from HPV negative HN-SCC and thus providing additional prognostic information; b) distinguish HPV positive metastases to the loco-regional lymph nodes derived from oropharyngeal cancers versus metastases of other origins [20,21]; c) furnish potentially useful indications for cancer treatment options; d) contribute to the differential diagnosis of rhino-pharynx undifferentiated carcinoma (WHO type I potentially related to HPV infection whereas Type II and III potentially related to EBV); e) provide valuable information for Head and Neck Cancer research.

The aim of this study is to demonstrate the relationship among p16 protein expression, HPV-DNA detection- and virus integration status into the host DNA in HN-SCC at different anatomical levels, i.e., oral cavity (OSCC) and oropharyngeal cavity (OPSCC). In this study p16-IHC has been used as the initial screening method followed by ISH/PCR to show the morphological context of HPV-DNA sequences detected by Consensus PCR.

## Results

### p16-IHC expression and promoter methylation of CDKN2a locus in HN-SCC

**Figure 1:**
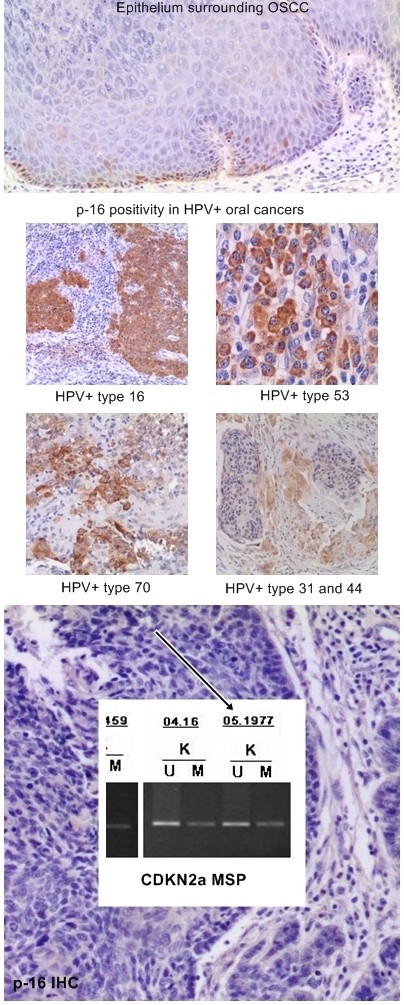
**IHC expression of p16 protein in representative HPV positive (DNA type 16) and HPV negative OSCCs**. P16 is expressed only at basal-parabasal levels in epithelium surrounding OSCC, whereas it is over-expressed in OSCC. The figure in the middle page depicted p16 expression according different HR and LR-HPVs. The figure in the bottom represent a p16 negative OSCC; the p16 under-expression is due to promoter methylation od CDKN2a. Legend. K, cancer samples; M, CDKN2a methylated; U, CDKN2a unmethylated.

**Figure 2:**
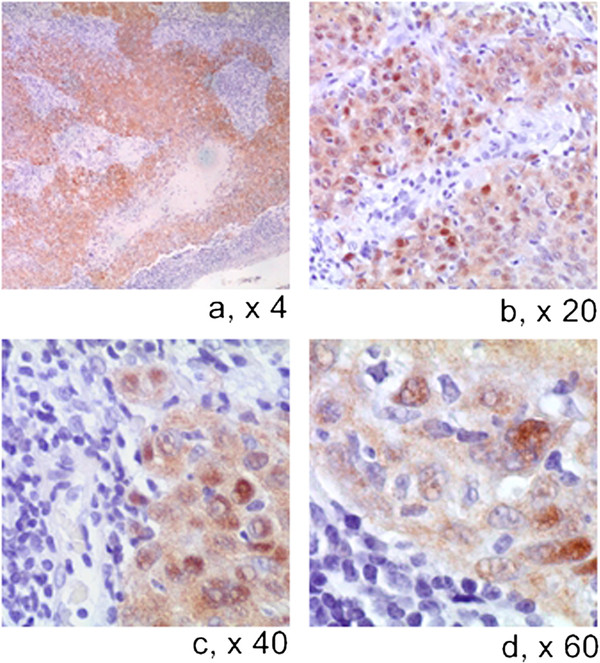
**IHC expression of p16 protein in OPSCC showed at increasing magnification**. Note intense nucleo-cytoplasmic expression (LSAB-HRP, nuclear counterstaining with haematoxylin).

**Figure 3:**
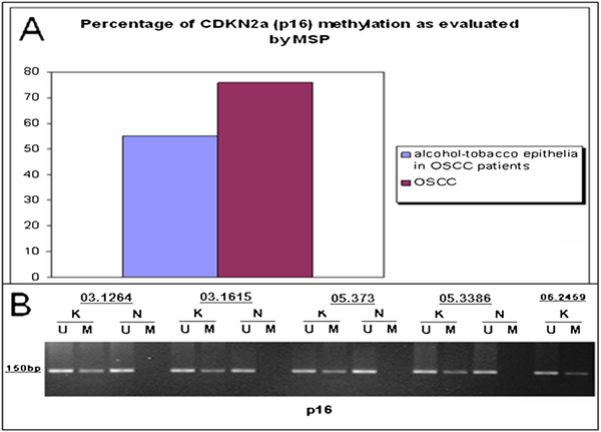
**The role of CDKN2a promoter methylation in p16 down-regulation**. A) Percentage of CDKN2a promoter methylation in OSCC and in oral epithelia exposed to alcohol and tobacco risk factors. B) Representative examples of Methylation-Specific PCR (MSP) for *CDKN2a/INK4a *locus (p16) in OSCC. Legend. K, cancer samples; N, normal samples; M, CDKN2a methylated; U, CDKN2a unmethylated.

p16-IHC expression has been evaluated in a total of 86 Head and Neck squamous cell carcinomas. In summary, 22 cases of OPSCC and 11 cases of OSCC have been evaluated using individual specimens by whole section method and 53 cases have been evaluated on TMA cores. P16 is normally expressed in restricted basal-parabasal layers of oral epithelium whereas it is over-expressed in OSCC and OPSCC (Figures 1 and 2). The protein expression in positive OSCCs was diffusely distributed in almost all cancer cells and localized in nuclei and/or cytoplasms. Remarkably, all the HPV positive cases by Consensus PCR analysis were also p16-IHC positive; in particular, also LR-HPV positive cases showed p16 protein expression in cancer cells although mainly distributed in cytoplasms. However, with the p16 antibody used in our study, the p16 sub-cellular localization by immunostaining was not predictive of HR-HPV detection as we observed HR-HPV type 16 positive OPCCs exhibiting cytoplasmic p16 staining. OPCCs with negative p16 staining were mainly observed in the patient cohort with a known history of alcohol-tobacco consumption and low HR-HPVs prevalence [22,23]. These latter cases were further analyzed by Methylation-Specific PCR and showed a high frequency (75%) of CDKN2a promoter methylation, which may explain the negative p16 protein expression (Figures 1 and 3).

### Analysis of HPV-DNA detection in controls

Fifteen cases of normal oral (5 cases), oropharyngeal ( 5 cases) and laryngeal (5 cases) specimens were negative for HPV-DNA by ISH assay using Inform HPV family-III (Ventana - Roche) and Inform HPV family-II (Ventana - Roche) and consensus primer PCR. We have also included in our study three control cases of HR-HPV positive cervical SCC lesions (two cases of HSIL - High grade Squamous Intraepithelial Lesion - and one case of invasive cervical carcinoma), along with one control case of Juvenile Onset Recurrent Respiratory Papillomatosis (JO-RRP) positive for low risk LR-HPV. All these controls cases were previously characterized for HPV status by PCR followed by direct sequencing.

### Combined HPV-DNA detection by ISH and PCR techniques

The combined HPV-DNA and ISH results are reported in Table 1.

**Table 1:** Interaction between HPV virus detection as evaluated by molecular methods and in situ hybridization signals.

Case	Year	Age-Sex	Italian Region	Site	HistologicalGrade	pTNM stage	In situ hybridization	Consensus PCR based method
**OPSCC-1**	2004	69 F	Puglia	Tonsil	G1	Biopsy	Negative	negative
**OPSCC-2**	2006	79 F	Puglia	Tonsil	G3	Biopsy	Negative	negative
**OPSCC-3**	2007	55 M	Puglia	Tonsil	G2	Biopsy	Negative	negative
**OPSCC-4**	ND	51 F	Puglia	Tonsil	G2	Biopsy	Negative	negative
**OPSCC-5**	2008	47 M	Puglia	Tonsil	G2	Biopsy	Negative	negative
**OPSCC-6**	2008	63 M	Puglia	Tonsil	G3	Biopsy	Negative	negative
**OPSCC-7**	2009	46 M	Puglia	Tonsil positive Uvula	G2	Biopsy	Negative	negative
**OPSCC-8**	2006	54 M	Puglia	Tonsil	G2	Biopsy	HR-HPV: nuclear clusters	negative
**OPSCC-9**	2008	45 M	Campania	Tonsil	G2	pT2NxM x	HR-HPV: integration positive clusters	HPV16
**OPSCC-10**	2007	60 F	Campania	Tonsil	G3	pT1NxM x	negative	HPV16
**OPSCC-11**	2007	62 M	Campania	Tonsil	ND	pT2NxM x	negative	HPV16
**OPSCC-12**	2007	51 M	Campania	Tonsil	G3	pT1NxMx	negative	negative
**OPSCC-13**	2008	67 M	Campania	Tonsil	G3	pT1NxMx	HR-HPV: clusters	HPV16
**OPSCC-14**	2006	55 F	Campania	Tonsil	G3	pTxNxMx	negative	negative
**OPSCC-15**	2009	69 F	Campania	Tonsil	G3	pT2NxMx	HR-HPV: focal clusters	HPV16
**OPSCC-16**	2008	79 F	Campania	Tonsil	G2	pT4aNxMx	negative	negative
**OPSCC-17**	2008	87 M	Campania	Tonsil	G3	pT1NxMx	HR-HPV: focal clusters	negative
**OPSCC-18**	2009	62 M	Campania	Tonsil	G3	pT4aNxMx	ND	HPV16
**OPSCC-19**	2009	66 M	Campania	Lingual tonsil - vallecula	G3	pT2N2bM0	negative	ND
**OPSCC-20**	2007	61 M	Sicilia	Tonsil	G3	pT2N0Mx	HR-HPV integrative	negative
**OPSCC-21**	2009	42 M	Sicilia	Tonsil	G2	pTxNxMx	negative	negative
**OPSCC-22**	2011	60 M	Sicilia	Tonsil	G3	pT2N0Mx	HR-HPV: focal nuclear clusters	ND
**OSCC-1**	1999	75 F	Marche	Tongue	G2	pT1N0M0	cytoplasmic signals	HPV31/44
**OSCC-2**	2000	59 M	Marche	Oral cavity (WRA)	G3	pT2N2bM0	negative (not available probes)	HPV 53
**OSCC-3**	2001	48 M	Marche	Tongue base (WRA)	G1	pT1N0M0	negative (not available probes)	HPV 70
**OSCC-4**	2002	63 M	Marche	Tongue	G3	pT2N1M0	ND	HPV 6
**OSCC-5**	1999	74 M	Marche	Oral cavity (WRA)	G3	pT2N0M0	HR-HPV: integration positive focal clusters	HPV 16
**OSCC-6**	2009	72 M	Sicilia	Oral cavity	G1	pT1N0M0	HR-HPV: faint focal nuclear clusters	HPV16/56
**OSCC-7**	2009	49 M	Sicilia	Tongue	G2	pT2N1Mx	negative	negative
**OSCC-8**	2010	41 M	Sicilia	Tongue	G2	pT1N0Mx	negative	negative
**OSCC-9**	2008	37 M	Sicilia	Hard palate	G1	pT2N0Mx	negative	negative
**OSCC-10**	2009	72 M	Sicilia	Oral cavity	G1	pT1N0M0	negative	HPV16/56
**OSCC-11**	2010	74M	Puglia	Tongue	G1	pT2N0M0	negative	ND
**TMA OSCC (53 cases)**	1997-2008	20 F/33 M	Campania	Oral cavity- multiple sites	All G available	All TNM available	negative	negative

The results are compared to clinic-pathologic parameters and demographic data

TMA: tissue microarray; ND: non determined data; F: female; M: male; WRA: Waldeyer Ring Area

### OSCC analysis

Out of the 64 total OSCCs cases analyzed by ISH, in only one case we were unable to diagnosed the infective status of the oral mucosa. Furthermore, we found 2 cases positive for HPV-DNA by Inform HPV family-III (Ventana - Roche) and no case positive for DNA by Inform HPV family-II (Ventana - Roche) with a total of 2 HPV positive oral cancers.

Our IHC results were confirmed by the Consensus PCR; similarly, we could not diagnosed the HPV infection status of the epithelium in only one case out of the total 64 OSCCs.

Furthermore, our Consensus PCR analysis performed on OSCCs indicated HR-HPV status not exclusively restricted to HPV 16. In more detail, out of the total 63 cases diagnosed as HPV positive by both IHC and Consensus PCR, we found three cases positive for HPV types 16 and 16/56 (which represents 4.76% of the HPV positive OSCCs). Furthermore, two additional HR-HPVs were found, the HPV types 53 and 31, the HPV 53 positive case was not valuable by ISH with our antibody, while the HPV 31 was found by ISH as non integrated. As regard with the LR-HPV types, the analysis by ISH alone was delusive because 2/3 (in particular, HPV 44 and HPV 70) (66.66%; SE ± 0.273) of LR-HPVs detected by PCR in our OSCCs collection were not included in the commercially available Inform HPV family-II probe Ventana - Roche.

### Concordance analysis of HPV-DNA detection by p16 immunhistochemistry and HPV-DNA molecular methods (ISH and PCR techniques) in OSCC

All HPV positive OSCCs showed p16-IHC positivity with high and diffuse levels of p16 immunostaining. Therefore, the p16 immunohistochemical method (p16-IHC) proven a rate of 100% sensitivity as a detection method of HPV positive OSCC cases with no false negative (p16 negative; HPV positive) cases. On the other hand, our results demonstrate that a specificity rate of 74%, with a false positive subgroup (26%) of OSCC cases which were p16-IHC positive but HPV-DNA negative.

### OPSCC analysis

By ISH, in only one case out of the total 22 individual OPSCCs sections(4.5%; SE ± 0.044) we analyzed, we could not determine the infection status of the epithelium. Out of the 21 HPV positive cases diagnosed by IHC, we found that 7 cases were also positive for HPV-DNA by Inform HPV family-III (33.3%; SE ± 1.103), but none was positive for DNA by Inform HPV family-II. Of these total of 7 cases found HPV positive OPSCCs by Inform HPV family-III, 6 were also positive by the PCR techniques.

By Consensus PCR, we were unable to determine the infection status of the oropharynx epithelium in only 2 cases out of the total analyzed 22 OPSCC (9%; SE ± 0.061). Six cases (30%; SE ± 0.102) out of the 20 positively diagnosed cases of OPSCC were PCR positive and all these six cases were type HPV16.

By using PCR method in combination with ISH we were able to detect four additional HR-HPVs ISH positive cases among the PCR-negative group (3 PCR-HPV-DNA negative and 1 PCR beta-globin negative on FFPE); with a final count of 10 HPV positive OPSCCs considering also single positivity by at least one DNA detection method. OPSCCs evaluated by ISH showed positive nuclear signals in 6/21 (28.5%; SE ± 0.099) with integrative punctuate stain detected in 2/21 cases (9.5%; SE ± 0.064). Consensus PCR detected 3 additional cases not detected by ISH (2 cases ISH-negative and 1 case ISH failed).

### Concordance analysis of HPV-DNA detection by p16 immunhistochemistry and HPV-DNA molecular methods (ISH and PCR techniques) in OPSCC

The p16-IHC method also proved a 100% sensitivity rate in OPSCC cases; all HPV positive OPSCCs showed p16-IHC positivity with high levels of p16 immunostaining. Its specificity rate of 93.5%, was much higher in OPSCC than in OSCCs, with a smaller false positive subgroup (of only 9%) in OPSCCs cases which were p16-IHC positive but HPV-DNA negative.

Overall, the agreement between the ISH and Consensus PCR techniques as methods of detection for HPV-DNA is shown in Table 2. It was not technically possible to perform the two test together in 3/22 OPSCCs (13.63%; SE ± 0.079) and in 2/64 OSCCs (3%; SE ± 0.021) which bring these to a total of only 5 HN-SCC cases (5.8%; SE ± 0.025) from the initial 86 cases included in our analysis. The tests were concordant in 14/19 OPSCCs (73.68%) and in 58/62 OSCCs (93.5%). Regarding oral malignancies (OSCC collection) the Fleiss' kappa coefficient (κ = 0.44) suggested moderate concordance according to the method of Landis and Koch [24]. As regards oropharyngeal malignancies (OPSCC collection) the concordance was fair (κ = 0.38) according to the above mentioned method.

**Table 2:** Agreement between ISH and Consensus PCR to detect HPV-DNA in OSCC and OPSCC

*ISH results Including LR and HR*	No. of cases examined by PCR (%)		Sensitivity	Specificity	K	Total observed agreement (ISH/PCR)	Total expected agreement (ISH/PCR)	
						
Diagnosis	*Negative*	*Positive*						
*Negative*	56(90.3%)	4(6.5%)	33.3%	100%	**0.44**	58(93.5%)	83%	
*Positive*	0(0%)	2(3.2%)						
								
*Negative*	11(57.9%)	2(10.5%)	60%	78.5%	**0.38**	14(73.7%)	58%	
*Positive*	3(15.8%)	3(15.8%)						
								

### Site by site distribution of HPV positive cells and HPV ISH signal patterns

#### In situ hybridization for HR-HPV in oral cancer (OSCC)

**Figure 4:**
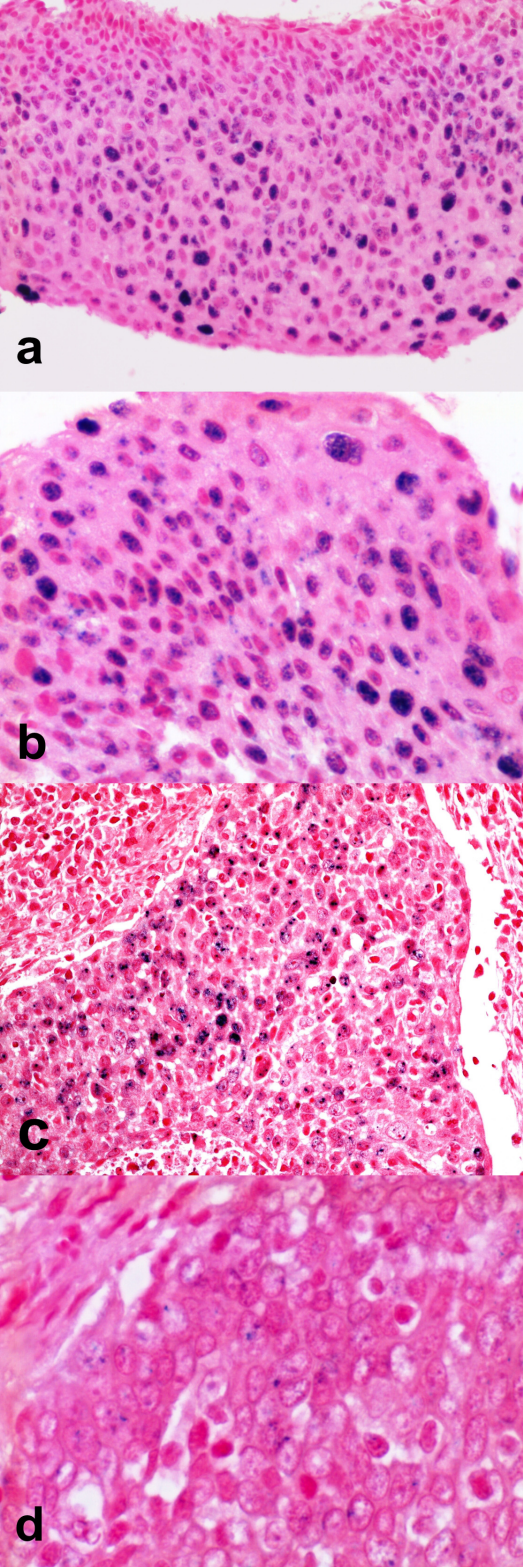
**HR HPV ISH in Head and Neck squamous cell carcinomas**. Note the clear nuclear staining with tetrazole blue as punctuate signals and clusters, corresponding to integration and episomic status respectively A, B) Control cases of cervical HSIL. C) Integration and episomal clusters are uniformly distributed in tonsil cancer (OPSCC) harboring type 16 HPV-DNA; note integrative punctuate signals and cluster spot throughout the entire tumor. D) Heterogeneous integration signals in oral cancer harboring type 16 HPV-DNA. (In situ hybridization; tetrazole blue signals stain the viral DNA; nuclear counterstaining with fast red; original magnifications a, b, ×10; c, ×40; d ×60).

**Figure 5:**
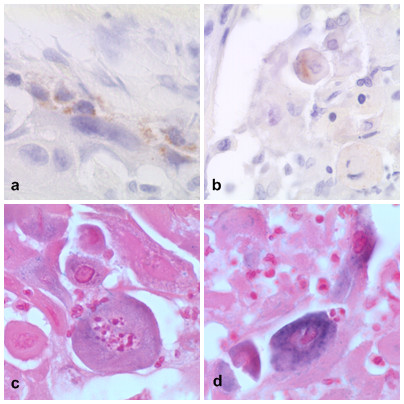
**ISH techniques for HPV show the morphological context of viral DNA location. Representative case of HPV-DNA positive tumor without virus integration in cancer cells**. In spite of positivity for two HPV viruses (HR-HPV 31 and LR-HPV 44 viruses) this case of OSCC showed no integration but cytoplasmic localization in cancer cells as evaluated by two commercially available in situ hybridization techniques; note the cytoplasmic virus location in autolytic cancer cells *(All the pictures in this panel were selected from different fields of a single case. A, x40), B, x40), DAKO ISH, brown DAB signals stain the viral DNA; C, x60), D, x60), Ventana ISH, tetrazole blue signals stain the viral DNA)*.

The OSCC case, HPV 16 positive by PCR, showed prevalent discreet dot spot signal of integration with only focal clusters and has been categorized as integrated (Table 1 and Figure 4). In a characteristic double infected oral cancer (HPV31 and 44), we have not observed a nuclear integration (Figure 5) using ISH, although the two HPV viruses have been detected by PCR. Unfortunately, it has not been possible to determine the integration status of HPV type 53 by PCR because probes for this HPV variant have not been commercially available nor have they been standardized for clinical laboratory use.

#### In situ hybridization for HR-HPV in oropharyngeal cancer (OPSCC)

Our study showed that 7 of 21 (33.3%; SE ± 1.103) of the OPSCCs, all of them originated from the tonsil region, were HPV positive when ISH was used as single test;. Interestingly, only 1 out of 7 cases in the female population was ISH positive (14.2%; SE ± 0.132) whereas we found 6 out of 15 cases in male population were ISH positive (40%; SE ± 0.126). With the addition of the Consensus PCR test all cases HPV positive by ISH demonstrated type specific restriction for the HR-HPV type 16. The distribution of integrative and episomal signals is shown in Table 1. One case showed the typical integrative (I) dot-spot signals as evaluated by ISH; one case demonstrated a mixed episomal-integrative (E-I) pattern, while the other 5 cases showed exclusively episomal pattern (E) of HPV-DNA as demonstrated by nuclear cluster observation.

Two cases were negative by ISH but HPV positive by PCR, while five out six PCR positive cases where also ISH positive. In one PCR positive case we could not establish the ISH result. Therefore, the ISH technique demonstrated a sensitivity of 60% when used in combination with PCR (see Table 2). We have observed three cases in positive agreement by ISH for HR-HPV and PCR for HR-HPV type 16, with a specificity of 78.5% for ISH technique when used in combination with PCR. This specificity rate has been obtained with a careful exclusion of false positive staining as described in the methods for evaluation.

### In situ hybridization for LR-HPV in OSCC and OPSCC and its relationship with haematoxylin and eosin coilocytosis detection and HPV-DNA techniques

Techniques directed to detect HPV-DNA in OSCC and OPSCC have shown different mucosal HPV low risk types (LR-HPV 6, LR-HPV 44, LR-HPV 70). According to our previous observations and to the current literature these low risk viruses may be detected alone or together with high risk viruses in multiple infection. Interestingly only squamous cell carcinomas of the oral and oropharyngeal cavities infected by LR-HPV showed coilocytosis as evaluated by classical histological haematoxylin and eosin staining. This explains the low power of coilocytes demonstration in detection of HPV-related Head and Neck Cancer since it can detect only single LR-HPV, associated to SCC but not causative of cancer, or double/multiple HPV infection including a LR-HPV. In a representative double infected oral cancer case (HR-HPV 31 and LR-HPV 44), by ISH we have observed neither nuclear integration nor nuclear clusters, but cytoplasmic localization in cancer cells although the PCR DNA method has revealed the effective presence of the two HPV viruses (See Figure 5). In other cases with LR-HPV positive HPV-DNA based methods, in situ hybridization was not applicable as the probes were not included in the commercially available probe sets we used; these commercial probes have been standardized to detect the most common HPV types currently recognized as etiologic factors in female genital carcinogenesis.

## Discussion

The p16INK4a gene (CDKN2a/INK4a) functions as negative regulator of the cell cycle progression through its inhibition of cdk4/6 and subsequent blockage of the cyclin-dependent phosphorylation of the Retinoblastoma gene (Rb). CDKN2a/INK4a located on 9p21 is frequently inactivated in oral epithelial pre-cancer and cancer via the following events: LOH, hypermethylation, deletion, mutation. The loss of p16INK4A expression defines a subgroup of oropharyngeal cancer patients with worse clinical outcome [16] whereas p16 protein over-expression has been proposed as surrogate marker of HPV infection initially in cervical cancer by Amortegui et al., [25,26], followed by squamous cell carcinomas from other sites than uterine cervix [27], and especially in Head and Neck Cancers [28,29].

In order to further explain differences between p16 expression and HPV-DNA in oropharyngeal cancer we performed Methylation-Specific PCR (MSP) to evaluate CDKN2a/INK4a promoter inactivation. Finally, MSP showed that the methylation of CDKN2a/INK4a is a frequent epigenetic alteration in oropharyngeal cancer and revealed that p16 was inactivated in 75% of OPSCCs associated to alcohol and tobacco risk factors. Therefore, we can conclude that OPSCC should be ideally subdivided in two groups: a) alcohol-tobacco-associated/HPV-DNA negative/CDKN2a-MSP methylated/p16-IHC negative or faint; b) alcohol-tobacco-unassociated HPV-DNA/HPV-DNA-positive/CDKN2a-MSP unmethylated/ p16-IHC positive. However, the situation in clinical setting is more complex because different risk factors frequently overlapped i.e. in HN-SCCs of young population, alcohol/tobacco consumption and HPV infection may be also associated. HPV positive OPSCC mostly occurs in younger patients and may also arise in people without a history of tobacco use. Furthermore, there are some cases showing discrepancy between promoter methylation and protein expression; i.e. cancer cases in which promoter is methylated (as evaluated by qualitative analysis) and the protein is unexpectedly expressed. Therefore, in these cases quantitative tests should be performed in order to establish the proportion of methylated/unmethylated alleles in the cancer cells clearly distinguishing the latter from alleles of non-cancerous cells. Although the microdissection-based quantitative tests are important research tools they cannot be easily performed in current clinical practice. Furthermore, we observed different levels of p16-IHC accuracy in the different cancer subpopulation studied. In details, in a cohort of prevalently alcohol/tobacco associated cancers from the south-west of Italy (Napoli) p16-IHC test showed a lower level of specificity in detecting HPV positive cases. In this cohort, there was an unacceptable large group of p16-IHC positive cancers that were diagnosed as negative by the combined ISH and PCR methods. It is possible that up-regulated p16 expression we observed in cancers was due to other molecular events not related to HPV. On the other hand, in a cohorts with higher number of HPV positive cases, i.e. the cohort from middle-east Italy (Ancona) the p16-IHC test increases its specificity in detecting HPV cases. This observation is confirmed by our analysis of OPSCCs cases which showed higher level of HPV infection than that of OSCC, in parallel with higher p16-IHC specificity levels as method of detection of HPV positive cases. In agreement with this hypothesis, a recent literature report demonstrates different p16 accuracy according to different anatomical sub-sites of the Head and Neck region [30]. In this complex scenario the p16-IHC test alone or in association to CDKN2a promoter methylation could be used only as a screening method and need to be associated with molecular tests in order to detect HPV-DNA and to assess its integration status.

Integration of HPV-DNA into the host DNA is a well known topic in cervical cancer but there are few investigations in Head and Neck Cancers. Integration of HPV 16 DNA correlates with dysfunction of HPV E1 or E2 open reading frames (ORF), which are active during HPV replication.. E2 loss of function allows up-regulation of E6 and E7 oncoproteins, because E2 is a repressor of E6 and E7. The great percentage of cervical cancers harbors HPV in the integrated form, however, recently has emerged that cervical cancers may contain HPV episomic DNAs as well. It has been also demonstrated that HR-HPV episomal DNAs up-regulate the activity of E6/E7 promoter, which in turn gives rise to elevated E6 and E7 protein expression in cancer cells. As regard to HN-SCC HPV-DNA integration there are numerous interesting points to discuss. On the one hand current literature describes the HPV-related HN-SCC as almost exclusively HPV type 16 restricted cancers [31] and HPV type 16 is considered to have the highest capability to integrate into the host DNA in cervical high grade squamous intraepithelial lesions (HSILs) and invasive carcinomas [32,33]. On the other hand, HN-SCCs, in particular tonsillar cancers (TCs), have been described as tumors with elevated frequency of HPV-DNA type 16 in integrated or episomal form producing E6 and E7 oncogenic proteins, since the early observations of Sniders PJF at al. [34]. These observations have been further confirmed by Mellin H. et al. concluding that in oropharyngeal cancers HPV is almost exclusively not integrated and its carcinogenic activity is due to E6/E7 oncoproteins expressed from episomal viral sequences [35]. Recently, some bias in the interpretation of HPV prevalence in HN-SCCs have been emerged since oral cancers (OSCCs) have not been clearly distinguished from oropharyngeal ones (OP-SCCs). Aim of this study was to demonstrate the integrative versus episomic HPV status as a diagnostic tool separating OSCC from OP-SCC in order to furnish site by site information regarding percentage and distribution of viral integration in host cancer cells.

For this purpose, the ISH technique for HPV is able to reveal the morphological context of viral DNA location. Our ISH study showed heterogeneous status of HPV integration in cancers originated from different Head and Neck regions. Among the HPV positive OSCCs only the case infected by HPV type 16 DNA showed clear integration signals, although not uniformly distributed throughout the entire tumor but restricted to some cancer fields. Other fields of the same tumor specimen showed clusters signals demonstrating episomal status. Therefore, the HR-HPV type 16 OSCC harbor both integrated and episomic HPV-DNA into the host transformed cells but with an heterogeneous distribution. Interestingly, the OSCC case with double LR-HPV type 44 and HR-HPV type 31 infection showed no signals of integration; however, the viruses have been localized exclusively in the cytoplasms of the cancer cells. Remarkably, we found a case of OSCC infected by the HPV type 53, recently included in a phylogenetic group of HR-viruses unrelated to HR-HPV 16 and HR-HPV 18 [36]. Unfortunately this case with type 53 HPV virus has been not determined by ISH because commercially available systems do not include this type 53 in the HR-HPV probe panel. This is because most detection systems for HPV detection are tailored for female genital system and not for other organ systems.

As regard to OPSCC we found the most HPV-DNA positive cases in tonsillar cancers (TCs). As described in literature HPV16 is the almost unique HPV-DNA found in this anatomical sub-site of the OP region. We demonstrated that TC cancers had intense integrative and clusters signals of HR-HPV diffuse throughout the whole cancer specimen and not focally distributed as we have observed in the OSCC where the integrative signals were focally restricted to some fields of the cancer specimen. This is a further demonstration that HPV 16 plays a significant role in the pathogenesis of a subgroup of OPSCC and that the HPV 16 virus integration into the host genome begins in the tonsillar cripts leading to indirect stimulation of CDKN2a locus and p16INK4a over-expression [37-39].

Schache AG et al. [40] assessed the sensitivity, specificity, and prognostic ability of eight possible assays and assay combinations for HPV16, including the gold standard of RNA qPCR, in 108 cases of OPSCC from the United Kingdom. The investigators found that HPV16-positive patients with OPSCC were younger and smoked less than HPV negative patients, and the proportion of HPV16-positive cases increased from 15% to 57% between 1988 and 2009. When compared to RNA qPCR, p16-IHC/DNA qPCR showed sensitivity and specificity of 97% and 94%, respectively, and proved to be the best discriminator of favorable outcome. The p16-IHC/HR-HPV ISH had a specificity of 90%, but reduced sensitivity of 88% affected the prognostic utility. Used in isolation, p16-IHC, HR-HPV ISH, or DNA qPCR were not specific enough to be recommended for use in clinical trials. In agreement with our findings, the authors recommended "caution [...] in applying HPV 16 diagnostic tests because of significant disparities in accuracy and prognostic value" [40].

## Conclusions

Although HR-HPV detection is of utmost importance in clinical setting of HN-SCCs there is no agreement about the 'golden standard' considering the number of molecular methods or combinations available. Quantitative E6 RNA PCR has been considered as the gold standard thanks to the very high accuracy and very high statistical significant association with prognostic parameters. Quantitative E6 DNA PCR has a very high level of accuracy but lower prognostic association with clinical outcome compared to E6 RNA PCR. However, while it is possible to perform quantitative PCR on FFPE samples the maximum accuracy is found using fresh frozen tissue. Furthermore, not all laboratories have the same ability to perform quantitative molecular methods on both FFPE and fresh tissues. Therefore, in clinical practice and according to the recently published Head and Neck Cancer international guidelines a p16-IHC test is currently performed. Our study has demonstrated that although p16-IHC is a good prognostic indicator when used in combination with HPV-DNA molecular methods, it is not satisfactory when evaluated as HPV detecting test as used alone (specificity less than 75%). Adding ISH, although it is a method known to be less sensitive than PCR based ones, has the advantage to preserve the morphological context of HPV-DNA signals in FFPE samples and, unexpectedly, increased the sensitivity of p16/Consensus PCR combination.

## Methods

### Population and study design

The study group was composed of 33 patients with SCC of the oral cavity (n = 11 cases with OSCC) or of the oropharynx (n = 22 cases with OPSCC)(see Table 1). To further increase the number of cases, a Tissue Microarray (TMA) composed of 53 OSCC cases from National Cancer Institute of Napoli, Italy, has employed. The study included also 15 negative control cases of normal oral (n = 5), pharyngeal (n = 5) and laryngeal (n = 5) mucosa; 3 control cases of cervical HR-HPV positive lesions (n = 2 cases of HSIL and n = 1 case of invasive cervical carcinoma), and 1 positive LR-HPV positive control case of Juvenile Onset Recurrent Respiratory Papillomatosis (JO-RRP), all of them previously characterised for HPV by PCR followed by direct sequencing. Informed consent was obtained from all participants or from their relatives. The histopathological diagnosis of OSCC/OPSCC was made at the Section of Anatomic Pathology of the University of Foggia - Foggia, Italy. Microscopic evaluation was performed by two oral pathologists (GP and PB) determining the degree of differentiation according to WHO grading system, and establishing tumor extent according to the TNM system [41]. The collection of representative cases of OSCCs and OPSCCs has been made available for this study through the active collaboration between multiple Italian Universities and Cancer Research Centres (University of Foggia, University of Napoli Federico II, Napoli National Cancer Institute, University of Palermo, University of Ancona). Consecutive cases have been randomly chosen from cohorts known for their HPV prevalence as previously published [22,23] and were analyzed for HPV-DNA using FFPE specimens obtained from surgery with curative intention; in addition some cases have been also analysed using cytological brushing materials in preoperative setting. In situ hybridization was performed using commercially available panels of LR-HPV and HR-HPV probe and automated chromogenic ISH detection for HPV-DNA on serial sections FFPE blocks. On consecutive serial section from FFPE p16 immunohistochemistry was performed using p16 monoclonal Ab and standard LSAB-HRP technique. Methylation-Specific PCR (MSP) on consecutive serial 20 micron sections was performed to study inactivation of p16CDKN2a locus.

### Immunohistochemistry to detect p16 expression (p16-IHC)

Four μm serial sections from formalin-fixed paraffin-embedded blocks were cut and mounted on poly-L-lysine coated glass slides. Immunostaining was performed by linked streptavidin-biotin horseradish peroxidase technique (LSAB-HRP). After sequential deparaffinization and rehydration, the slides were treated with 0.3% H_2_O_2 _for 15 min to quench endogenous peroxidase. Antigen retrieval was performed by microwave heating - a 1^st ^time for 3 min at 650 W, a 2^nd ^and a 3^rd ^time at 350 W - of the slides immersed in 10 mM citrate buffer pH 6. After microwaving, the sections were blocked for 60 min with 1.5% normal horse serum (Santa Cruz Biotechnology, Santa Cruz, CA) diluted in PBS buffer before the reaction with primary antibody (Ab). Primary monoclonal anti-p16 antibody (BD-Pharmingen; clone G175-405) was diluted 1: 150 with 0.05 M Tris-HCl buffer pH 7.4 containing 1% bovine serum albumin and incubated overnight. After two washes with PBS, the slides were treated with biotinylated species-specific secondary antibodies and streptavidin-biotin enzyme reagent (DAKO, Glostrup, Denmark), and the color developed by 3,3'- diaminobenzidine tetrahydrochloride chromogen solution (DAB). Sections were counterstained with Mayer's haematoxylin and mounted using xylene-based mounting medium. Negative control slides without primary antibody were included for each staining. The results of the immunohistochemical staining were evaluated separately by two investigators. Stained cells were counted in at least 10 high powered (40×) fields using an Olympus BX41 microscope. For each case, the cumulative percentage of positive cells among all sections examined was determined.

### Tissue microarray based immunohistochemistry

For tissue microarray construction, areas of interest rich in non-necrotic tumor cells, were identified on corresponding haematoxylin and eosin-stained sections and marked on the source paraffin block. The source block was cored and a 0.6 mm core transferred to the recipient master block using Galileo TMA CK 3500 Tissue Microarrayer; ISE TMA Software (Integrated System Engineering, Milan, Italy). Two cores from different areas (one representative of superficial and one deep invasion) and whenever possible one core of normal mucosa of the same tissue block were arrayed for each case. All the donor cores were formatted into one recipient block. H&E staining of a 4-μm TMA section was used to verify all samples. Immunohistochemical analysis on 4-μm TMA serial sections was performed by using Ventana Benchmark^® ^autostainer and/or manual standard linked streptavidin-biotin horseradish peroxidase technique (LSAB-HRP), according to the best protocol for each antibody tested in our laboratory.

### PCR analysis

HPV-DNA was detected using nested PCR (MY/GP primers), and HPV genotype was determined by direct sequencing of PCR fragments. Three types of control were included in each reaction series: blank control, HPV negative Wi cells as negative control and HPV-18 DNA-positive HeLa cells, in dilutions from 20,000-50,000 down to 2-5 HPV-DNA copies, as positive control. HPV-DNA was amplified by PCR assay using primers useful for samples with a low copy number of HPV (MY09-MY11 primer pair in combination with GP5-GP6 primer pair) as previously demonstrated [42] and amplifications were performed in a Mastercycler gradient thermal cycler (Eppendorf, Hamburg, Germany); amplification products were analyzed in 8% polyacrylamide gel.

### Sequencing analysis

HPV genotyping was based on direct sequencing of MY or MY/GP PCR fragments. Amplification products were purified by Microcon YM-100 (Amicon-Millipore, Billerica, MA); the sequence of both DNA strands was determined by the BigDye Ready Reaction Kit in the automatic sequencer ABI Prism 310 Analyzer (both from Perkin-Elmer Applied Biosystems, Foster City, CA). Alignments were obtained from the GenBank on-line BLAST server and HPV sequences downloaded from the HPV database http://hpv-web.lanl.gov.

### In situ hybridization (ISH) for HPV-DNA detection in morphological context

ISH signal patterns has been reported to be associated to the status of HPV showing the test the power to detect episomal, integrated, or mixed forms [43,44]. Recently, a commercially available probe set for HR-HPV has showed an agreement with Consensus PCR of 85% in FFPE tissue specimens from patients with cervical intraepithelial neoplasia and cervical carcinoma [45]. This A commercially available HPV ISH system has been used (Ventana Inform HPV, Tucson, AZ, USA) to detect integration or episomic status in our study. Briefly, in situ hybridization was performed using the Benchmark^® ^plate and an alkaline-phosphatase coupled antibody detection method. The hybridization signals were shown with Tetrazole Blu and Fast Red nuclear counterstaining. The commercially available Ventana kit includes the following probes for HR-HPVs 16, 18, 31, 33, 35, 39, 45, 51, 52, 56, 58, e 66 (Iiform HPV family-III 16 Probe; Ventana - Roche); and the following probes for LR-HPVs, 6, 11 (Inform HPV famly- II 6 Probe; Ventana - Roche).

### ISH evaluation

ISH signals were determined for at least 10 high powered fields as described above. OSCC/OPSCC cases showing prominent nuclear punctuated (discreet dot-like) signals have been considered as integrative (I). Cases with exclusive nuclear cluster signals as been evaluated as episomal (E). The episomal pattern appeared as large, homogeneous, globular navy-blue precipitate within the cell nucleus. Recently it has been suggested that the signals originating from integrated virus can be hidden in a background of episomal HPV [46]; therefore cases showing a prevalent nuclear cluster signals along with also focal punctuated signals of integration have been evaluated as mixed episomic-integrative (E-I). According to the manufacturer, artifacts or non-specific staining is regarded as: non-cellular stromal precipitates; cytoplasms of PMNs (polymorphonucleated cells), eosinophils, lymphocytes and endothelial cells; and staining of nucleoli

### Methylation-specific PCR (MSP) to analyze promoter methylation of CDKN2a/INK4a locus

Following careful examination of Haematoxylin-eosin stained slides, we selected tissue sections with the greatest proportion of malignant tissue. Paraffin blocks with corresponding normal epithelium distant from tumor were as selected. Five 10 μM sections were cut from each formalin-fixed, paraffin-embedded tumor sample and transferred into micro centrifuge tubes. The paraffin was dissolved using xylene followed by two washes with 100% ethanol and one wash with phosphate-buffered saline. The samples were then incubated in lysis solution (proteinase K - Qiagen, 20 mg/ml, 50 micro-L; 1 M Tris HCl solution, 10 micro-L; 0,5 M EDTA, 2 micro-L; 10% SDS 100 micro-L; distilled water 838 ml) overnight at 55°C. Reversal of cross-linking was performed by adding NaCl (final concentration 0.7 M) and incubating at 65°C for 4 h. DNA was recovered using the Wizard DNA clean-up kit (Promega, Madison, WI) according to the manufacturer's protocols. To test the integrity of isolated DNA the housekeeping haemoglobin gene was amplified by PCR and visualized by gel electrophoresis for both control and pathological samples. The haemoglobin gene primers used were: *forward*, 5'-GAA GAG CCA AGG ACA GGT A-3', and *reverse*, 5'-GGA AAA TAG ACC AAT AGG CAG 3'. The DNA quantity was evaluated by a NanoDrop Spectrophotometer (CELBIO). Sodium bisulfate modification of 100 μg DNA for each sample was performed using the EZ DNA Methylation Kit (Zymo Research, Orange, CA) following the manufacturer's protocol, with the addition of a 5 min initial incubation at 95°C prior to addition of the denaturation reagent. The steps to reverse cross-linking in the extraction procedure as well as the 95°C incubation ensure more complete melting of the DNA and thus more complete sodium bisulfite conversion of these formalin-fixed specimens. All Methylation-Specific PCRs were optimized to detect > 5% methylated substrate in each sample. Each experiment was performed in triplicate. Methylated and unmethylated DNA were equally recovered from fixed material and only non-degraded DNA samples were selected. The primers used for Nested-PCR to flank methylated/unmethylated (M/U) *CDKN2a/INK4a locus *have been reported in Table 3.

**Table 3:** Primers used to detect methylated and unmethylated p16-CDKN2a locus by Nested PCR (MSP)

P16 EF	AGAAAGAGGAGGGGTTGGTTGG
P16 ER	ACRCCCRCACCTCCTCTACC
P16 IMR	GACCCCGAACCGCGACCGTAA
P16 IMF	TTATTAGAGGGTGGGGCGGATCGC
P16 IUR	CAACCCCAAACCACAACCATAA
P16 IUF	TTATTAGAGGGTGGGGTGGATTGT

### Statistical analysis

The data were analyzed by the Stanton Glantz statistical software 3 (MS-DOS) and GraphPad Prism software version 4.00 for Windows (Graph Pad software San Diego, CA, http://www.graphpad.com).

### Reliability

The HPV 'positivity' as evaluated by different methods was assessed using Kappa statistics. The reliability of ISH and Consensus PCR was determined using Fleiss' intra-class correlation coefficient (ICCC) [47,48]. The Kappa coefficients were divided into categories as described by Landis and Kock [49].

## Abbreviations

HPV: Human papilloma virus; HN-SCC: Head and neck squamous cell carcinoma; IHC: Immunohistochemistry; DNA: Desoxyribonucleic acid; PCR: Polymerase chain reaction; ISH: In situ hybridization; OSCC: Oral squamous cell carcinoma; OPSCC: Oro-pharyngeal squamous cell carcinoma; MSP: Methylation-specific PCR; FFPE: Formalin fixed paraffin embedded; RNA: Ribonucleic acid; USA: United States of America; Rb: Retinoblastoma protein; LOH: Loss of heterozygosity; WHO: World Health Organization; EBV: Epstein barr virus; TMA: Tissue micro-array; LR-HPV: Low risk human papilloma virus; HR-HPV: High risk human papilloma virus; I: Integrative pattern; E-I: Episomal-integrative pattern; E: Episomal pattern; ORF: open reading frame; TCs: tonsillar cancers; QPCR: Quantitative polymerase chain reaction; JO-RRP: Juvenile onset recurrent respiratory papillomatosis; LSAB-HRP: Linked streptavidin-biotin horseradish peroxidase technique; DAB: 3,3'- diaminobenzidine tetrahydrochloride chromogen solution; HPF: High power field analyzed; ICCC: Intra-class correlation coefficient.

## Competing interests

The authors declare that they have no competing interests.

## Authors' contributions

PG, SA, CG, RC, PS carried out the molecular studies, participated in the sequence alignment and drafted the manuscript. PMC, CS carried out the immunoassays. FR, BG, AG participated in the sequence alignment. RV, LLM participated in the design of the study and SA performed the statistical analysis. PG; SA PB, SS conceived of the study, and participated in its design and coordination and DRG, TML, BFM have made contributions to acquisition, analysis and interpretation of data and helped to draft the manuscript. All Authors read and approved the final manuscript.
